# Ensuring Food Safety for Americans: The Role of Local Health Departments

**DOI:** 10.3390/ijerph19127344

**Published:** 2022-06-15

**Authors:** Gulzar H. Shah, Padmini Shankar, Vinoth Sittaramane, Elizabeth Ayangunna, Evans Afriyie-Gyawu

**Affiliations:** 1 Department of Health Policy and Community Health, Jiann-Ping Hsu College of Public Health Georgia Southern University, Statesboro, GA 30460, USA; ea06435@georgiasouthern.edu; 2 School of Health & Kinesiology, Georgia Southern University, Statesboro, GA 30460, USA; pshankar@georgiasouthern.edu; 3 Department of Biology, Georgia Southern University, Statesboro, GA 30460, USA; vsittaramane@georgiasouthern.edu; 4 Department of Occupational and Environmental Health & Safety, School of Public Health, College of Health Sciences, Kwame Nkrumah University of Science & Technology, Kumasi 00000, Ghana; evans.afriyie-gyawu@knust.edu.gh

**Keywords:** food safety, local health departments, food inspection, primary prevention, food safety policy

## Abstract

(1) Background: Several agencies in the United States play a primary role in ensuring food safety, yet foodborne illnesses result in about 3000 deaths and cost more than USD 15.6 billion each year. The study objectives included analyzing local health departments’ (LHDs) level of engagement in food safety and other related services, and LHDs’ characteristics associated with those services. (2) Methods: We used data from 1496 LHDs that participated in the 2019 National Profile of Local Health Departments Survey, administered to all 2459 LHDs in the United States. Logistic regression analyses were performed to model multiple dichotomous variables. (3) Results: An estimated 78.9% of LHDs performed food safety inspections, 78.3% provided food safety education, 40.7% provided food processing inspections, and 48.4% engaged in policy and advocacy. The odds for LHDs to directly provide preventive nutrition services were 20 times higher if the LHDs had one or more nutritionists on staff (Adjusted Odds Ratio or AOR = 20.0; Confidence Interval, CI = 12.4–32.2) compared with LHDs with no nutritionists. Other LHD characteristics significantly associated with the provision of nutrition services (*p* < 0.05) included population size, state governance (rather than local), and LHD having at least one registered, licensed, practical, or vocational nurse. The odds of providing food processing services were lower for locally governed than state-governed LHDs (AOR = 0.5; CI = 0.4–0.7). The odds of performing food safety inspections varied by LHD’s population size, whether a nutritionist was on staff, whether it was state-governed (vs. locally), and whether it completed a community health assessment (CHA) within 5 years. (4) Conclusions: LHDs play a critical role in ensuring safe food for Americans, yet variations exist in their performance based on their specific characteristics. Adequate funding and a competent workforce are essential for LHDs to utilize evidence-based practices and engage in policymaking and advocacy concerning food safety.

## 1. Introduction

Food safety [1] refers to the conditions and practices that preserve the quality of food to prevent contamination and foodborne illnesses [2]. According to the World Health Organization (WHO), food is a key transmission channel for more than 200 diseases in people. Foodborne illnesses constitute a significant but largely preventable public health issue in the United States. Food contaminated with pathogenic microorganisms can cause serious illness, thereby impacting one’s quality of life and creating a preventable burden of disease [1]. According to the Centers for Disease Control and Prevention (CDC), since 2006, there have been several outbreaks of foodborne illnesses caused by major pathogens such as Salmonella species (93), *E. coli* (45), Listeria species (24), and other agents (Cyclospora-9; Hepatitis A-4; Vibrio sp-2 and Norovirus-1) [3]. The CDC estimates indicate that 17% of the U.S. population (i.e., 48 million people) get sick because of these illnesses every year, resulting in 128,000 hospitalizations and 3000 deaths [2]. The healthcare costs associated with foodborne illnesses are enormous, with the USDA estimating it to be almost USD 17.6 billion annually [4]. In a significant effort to reduce the health and economic burden, the U.S. Department of Health and Human Services has unveiled specific baseline and developmental goals on safe food handling in the Healthy People 2030 Initiative [5].

While the significance of keeping food safe is well understood, achieving food safety for all Americans is a complex undertaking. Each point along the farm-to-fork continuum has its unique challenge [6,7]. Food can get contaminated during production, processing, distribution, or preparation. Since food products pass through various steps such as production, processing, shipping, and marketing before reaching consumers, several factors serve as food safety threats [6]. Multiple intermediate handlers can introduce potential contamination threats, which necessitates the involvement of different agencies for continuous monitoring to ensure food safety [6,7,8]. Thus, food safety is a shared responsibility among all entities involved in producing, processing, shipping, selling, regulating, and consuming food [9,10].

In the United States, various organizations are involved in devising, implementing, and enforcing food safety policies at the federal, state, and local levels [11,12,13]. Consequently, seamless partnerships between all entities are critical for ensuring food safety [12,13]. While federal agencies are key food policy regulators, LHDs are primarily responsible for conducting foodborne illness prevention activities, inspections of food processing and food service establishments, and food safety education [14]. Variation may exist in LHDs’ engagement in the provision of such services, depending on their type of governance relative to state health agency authority configured as a centralized, decentralized, mixed, or shared structure [15,16,17,18].

Primary prevention activities for which LHDs are responsible involve foodborne illness surveillance and outbreak investigation [19]. Whereas foodborne illness surveillance is essential for efficient detection of disease clusters and potential problems in the food supply chain, outbreak investigations are critical in identifying the pathogenic agent(s), natural toxins, environmental chemical contaminants and pesticides, drug residues, physical contaminants, people at risk, mode of transmission, source(s) of contamination, the potential for further transmission, and disease control measures. LHDs play a key role in the inspection of food processing and food service establishments at specific intervals to collect food samples, examine equipment, and review records on the food and supplies used at the establishment [20]. It is also important to record any violations of the food safety regulations [21]. Further, LHDs are on the frontlines of food safety education and training [22]. LHDs are responsible for training employees and operators on safe food handling practices, informing communities about outbreaks and food recalls to prevent the widespread occurrence of foodborne illness, and disseminating health information on safe food practices related to food procurement, transportation, handling, preparation, and storage [23,24].

New and emerging food safety issues pose serious threats to LHDs facing budget constraints due to underfunding, [25] limited and untrained workforce personnel, [23] lack of coordination between state and federal agencies, inadequate research funding, and ineffective mechanisms to integrate science-based applications into food safety practices [12,26,27]. LHDs try to counter some of these challenges through evidence-based practice, use of health informatics, and employment of a diverse workforce of public health professionals, including nurses, epidemiologists, environmental health specialists, nutritionists, and health educators, who are the backbone of the infrastructure of LHDs [23,25]. 

Appropriate education and skill set of a competent workforce are essential for LHDs to deliver public health services associated with food safety. However, it is reported that a majority of public health workers lack formal public health training [23]. While the role of public health nurses in LHDs in providing health education to manage and control diseases in the community has been well studied [20,26], scarce evidence exists on the role of food and nutrition personnel in identifying, managing, and preventing foodborne illness. LHDs are important providers of food and sanitation services, [28] food safety inspections, [28,29] and food handler training programs [30]. Studies have highlighted the importance of regular food safety surveillance and education programs by LHDs in reducing foodborne illness [29,30]. Studies show that an inadequate public health workforce specially trained in food safety is the primary reason for the inability of several LHDs in the country to provide effective and sustained food safety-related services to the community [31,32].

Research evidence is scant regarding LHDs’ role in food safety policymaking, provision of nutrition services, food processing inspection, food safety inspections, and food safety education. The specific characteristics of LHDs that are either barriers or facilitators in providing these services have also not been studied recently. To contribute to the practice-relevant evidence, this study aims to describe LHDs’ engagement in food safety and policy activities, and analyze LHD characteristics associated with those activities.

## 2. Materials and Methods

### 2.1. Data

This study uses a retrospective, cross-sectional, quantitative survey study design. The study was exempted from a full review by the Institutional Review Board (IRB) of Georgia Southern University. Our study uses the 2019 National Profile of Local Health Departments (Profile) survey data collected by the National Association of County and City Health Officials (NACCHO) as their ninth survey of the Profile study since 1989–1990. The Profile study is a comprehensive source of data covering topics such as the LHD workforce, funding, leadership, governance, public health practice, policy and advocacy engagement, and other public health infrastructure and best practices [33]. The National Profile survey instrument, codebook, and data are available on request from the NACCHO website [33]. The 2019 Profile was administered to all 2459 LHDs meeting NACCHO’s definition of a local health department, with an overall response rate of 61%.

### 2.2. Measures

Five variables were used for gauging LHDs’ engagement in food safety services, primary prevention concerning food, and engagement in food safety-related policy and advocacy activities. The five variables of interest are (1) population-based primary prevention activity in nutrition, (2) inspection of food processing services, (3) inspection of food establishments, (4) food safety education and policy, and (5) advocacy activities in food safety. The variables LHD population-based primary prevention activity in food safety was measured by asking LHDs if they had directly performed population-based primary prevention activities [3] concerning nutrition in their jurisdiction during the past year, with options of either yes or no. The variables of LHD-provided inspection of food processing services and LHD-provided inspection of food service establishments were operationalized by the survey questions that asked if LHDs had provided inspection activities or services for food processing and food service establishments in their jurisdiction during the past year, with response options of yes or no for each of these inspections. The variables of LHD-provided food safety education and LHD engagement in policy and advocacy activities in food safety were operationalized by the survey questions that asked LHDs if they had provided food safety education in their jurisdiction during the past year and if they had been actively involved in food safety policy or advocacy activities in their jurisdiction during the past two years, respectively. Both variables had a dichotomous response option of either yes or no.

The multivariate analyses included LHD infrastructural and other characteristics potentially associated with these food safety activities. The variable representing the scope/scale of the LHD was captured through population size in the jurisdiction, grouped as <50,000, 50,000–499,999, and 500,000 or larger population. The other important LHD organizational characteristics were reflected through governance category (state governed, shared governance, local governance), whether the LHDs had performed community health assessment (CHA) with three response options of yes within 5 years, no, but plan to next year, and no CHA or not within 5 years. For the variables LHD employs one or more occupational nutritionists, the top executive has certification in public health, and LHD has a registered, licensed practical or vocational nurse, the response options were yes and no. 

### 2.3. Analytical Methods

We computed weighted percentages to show descriptive characteristics of the LHDs. To model five dichotomous dependent variables, five separate logistic regression models were computed to assess the association between the LHD engagement in food safety activities and other LHD characteristics described earlier. To account for the disproportionate response rate by LHDs’ sizes, survey weights developed by NACCHO were used in these analyses. All analyses were conducted using SPSS 25 [34] with proper statistical weights to account for the sampling design of the Profile and disproportionate response rates by size of the population in the LHD jurisdiction.

## 3. Results

**Figure 1:**
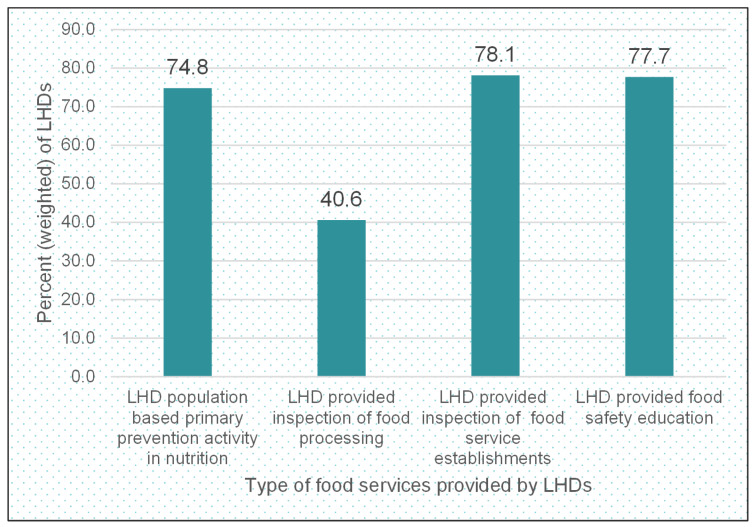
Percent of LHDs by type of food safety and other related services provided directly by local health departments. 2019 Profile of LHDs.

Inspection activities of food processing establishments were carried out directly by 40.6% of LHDs, and 2.1% of LHDs had these activities contracted out (Figure 1). 

Over 78% of LHDs directly provided inspection of food service establishments. The population-based primary prevention activities in nutrition were carried out directly by 74.8% of LHDs. To support food safety education, about 77.7% of LHDs provided relevant services directly. Roughly one in 2 LHDs (48.4%) engaged in policy and advocacy activities concerning food safety. A large majority of LHDs in the country are small or medium, with 55.9% comprising jurisdictions of <50,000 people and 36.9% serving the jurisdiction of 50,000–499,000 people. Only 7.2% have a population jurisdiction of 500,000 or more people (Table 1). 

The majority (69.7%) of LHDs are locally governed, 21.1% are governed centrally by a state public health agency, and 9.2% have shared governance functions between state and local authorities. LHD workforce statistics show that only 19.4% have an information system specialist on staff, 75.6% employ one or more occupational environment workers, 51% have occupational nutritionists, and 93.9% have at least one nurse. Only a small proportion of LHD top executives (20.9%) have a postbaccalaureate certificate in public health. LHDs’ engagement in strategic prioritization is evident from their heavy engagement in strategic prioritization in the past 5 years, with 79.4 % completing a CHA, 71.7% a CHIP, and 65.4% a strategic plan.

### 3.1. Inspection of Food Processing Services and Food Establishments

After the other variables in the logistic regression were controlled for (Table 2), LHDs’ governance type was the only variable significantly (*p* ≤ 0.05) associated with inspections of food processing services. The odds of provision of inspections of the food processing services were much lower for LHDs with local governance (AOR = 0.5; CI, 0.4–0.7) or shared governance (AOR = 0.6; CI, 0.4–0.9) than state governed LHDs. LHDs had significantly lower odds (*p* ≤ 0.05) of providing inspection of food establishments if they were governed locally (vs. State) (AOR = 0.4 CI, 0.2–0.6) or had not completed a CHA within 5 years but planned to conduct one in the next year (vs. did not complete or plan to complete CHA in the next year). When compared with smaller LHDs (i.e., those with <50,000 people in their jurisdiction), LHDs with population sizes of 50,000–499,999 had higher odds (AOR = 2.6; CI,1.9–3.6) of providing inspections of food establishments. The odds were also higher if LHD employed a nutritionist (AOR = 1.4; CI, 1.0–1.8).

### 3.2. Population-Based Primary Prevention Activity in Nutrition

Variables representing the type of workforce were among the most significant predictors of LHD engagement in population-based primary prevention activity in food and nutrition. The odds were higher for LHDs that employed an occupational nutritionist (AOR = 20; CI, 12.4–32.2) or had a registered, licensed practical or vocational nurse (AOR = 4.1 CI, 2.1–8.2). In contrast, the odds were lower for engaging in population prevention activity (AOR = 0.6; CI, 0.4–0.9) if the LHDs had an executive with certification in public health (vs. no certification in public health).

LHDs that had completed a CHA within the past five years had twice the odds (AOR = 1.9 CI, 1.2–2.9) of carrying out primary prevention activities in nutrition than LHDs that did not complete or planned to complete a CHA in the next year. Compared with the LHDs with population sizes of <50,000 in their jurisdictions, those with 500,000 or more people had significantly (*p* < 0.05) higher odds (AOR, 2.7; CI, 1.00–7.27) of carrying out primary prevention activities in nutrition. In contrast, LHDs with local and shared governance (vs. state governance) had significantly lower odds of carrying out primary prevention activities in nutrition (AOR = 0.2; CI, 0.1–0.3 and AOR = 0.3; CI, 0.2–0.8, respectively). 

### 3.3. Food Safety Education

Local health departments had higher odds of providing food safety education if their population size was between 50,000 and 499,999 rather than <50,000 (AOR = 1.7; CI, 1.3–2.3) or if they employed a nutritionist (AOR = 1.4; CI, 1.0–1.9). Odds of providing food safety education were lower for locally governed LHDs than state governed (AOR = 0.7; CI, 0.5–0.98) and for LHDs that did not complete community health assessment with an AOR = 0.4; CI, 0.2–0.7 but planned to complete it in the next year (vs. LHDs that did not complete CHA and had no plan to do so within the next year).

### 3.4. Policy and Advocacy Activities in Food Safety

The likelihood of engagement in policy and advocacy activities concerning food safety was significantly higher if the LHD was governed locally (vs. State) with an AOR of 2.8 (CI, 2.1–3.8). The odds were also greater if the population in LHD jurisdiction was 50,000–499,999 people (AOR = 1.7; CI, 1.3–2.2) or greater than 500,000 people (AOR = 2.6; CI, 1.6–4.1), rather than smaller population (<50,000 people). The odds were lower if the LHD had completed the CHA within the past five years (AOR = 0.6; CI, 0.4–0.8) or planned to complete one in the next year (AOR = 0.4, CI, 0.2–0.8) compared with LHDs that did not complete CHA and had no plan to complete it in the next year.

## 4. Discussion

While LHDs are key in preventing major foodborne outbreaks such as Salmonella, E. coli, and Listeria, there is a dearth of studies focusing on LHDs’ impact on food safety. The activities carried out by LHDs to ensure food safety vary by several organizational characteristics. The purpose of this study was to examine the level of LHDs’ engagement in ensuring food safety for people in their respective jurisdictions and evaluate LHDs’ characteristics associated with those activities. Our study demonstrated that the size of the population served, governance structure, public health certification of top executives, and employment of a nurse, nutritionist, and environmental health officer were associated with LHDs’ degree of engagement in carrying out activities related to food safety. These activities include inspection of food processing and food service establishments, provision of food safety education, and primary prevention concerning nutrition. Some of these activities are integral components of the Hazard Analysis and Critical Control Points (HACCP)—a systematic approach to the identification, evaluation, and control of food safety hazards [35].

The central finding of our study concerns the impact of having employees in certain functional categories of food safety and related activities, with the implication that the workforce capacity in specific functional categories (such as nutritionists and nurses) can be consequential in LHDs’ performance of food safety-specific public health services. Our study shows that LHDs with at least one occupational nutritionist on staff were more likely to provide primary prevention nutrition activities, inspection of food service establishments, and food safety education. This finding highlights the importance of having occupational nutritionists in the LHD workforce, given that these grassroots agencies are primarily responsible for inspecting food establishments in communities across the U.S. to ensure sanitation standards compliance and provide food safety education activities for food workers and the public. Since nutrition plays a critical role in the prevention and management of chronic diseases, there is a need for nutritionists in LHDs to deliver prevention activities targeted at risk reduction. To this end, a multidisciplinary approach that integrates basic nutrition education competencies in the public health curriculum should be considered [2]. Our study also showed that LHDs that employed professional nurses were more likely to implement food-related population-based primary prevention activities. However, the provision of other food safety and policy activities was not significantly affected by employing a nurse in the LHD workforce.

The economies of scope and scale positively affected LHDs’ involvement in food inspection, education, primary prevention, and policy activities, which are consistent with LHDs’ other activities and capacities, including cross-jurisdictional sharing [36], engagement in evidence-based practice [37], and electronic exchange of information [38]. For instance, we found that in comparison with smaller LHDs, those serving a population size of 50,000 to 499,999 were significantly more likely to engage in the inspection of food establishments and policy/advocacy activities and provide food safety education. LHDs serving larger populations may have the infrastructural capacities and workforce availability to effectively engage in these activities. LHDs in the largest population (500,000+) in their jurisdiction were significantly more likely to engage in policy and advocacy activities, for which the smaller LHDs might lack functional capacities. Resource availability may be a driving factor for the scope of services offered by LHDs [28]. In addition to the direct provision of workforce training and program-specific funding to the smaller LHDs, resource and information-sharing may resolve issues concerning the lack of capacities in scale-deficient LHDs [36]. 

LHDs’ governance type relative to state health agency authority presents an interesting variation in food safety and policy engagement. LHDs with local governance were less likely than state-governed LHDs to perform population-based primary prevention activities concerning food, the inspections of food processing and food establishments, and the provision of food safety education. Surprisingly, locally governed LHDs were more likely to engage in policy and advocacy activities in food safety. These findings indicate that although locally governed LHDs may need capacity building in other food-safety activities, it is state-governed LHDs that may need awareness raising, training, skill development, and strategic prioritization in policy and advocacy concerning food safety.

LHDs headed by top executives with certification in Public Health but without any formal degree were less likely to implement food safety activities. In this era of evidence-based practice that seeks to eliminate health inequities, higher-level administrators with a public health professional education are vital to reducing food safety risks among Americans. This is consistent with a multicounty study that found that many health care professionals do not have formal education and training in public health but are just certified, which makes them less equipped to engage in foodborne illness prevention activities [39]. 

This study’s findings and conclusions should be interpreted within their limitations. Although the study used a census design and the Profile survey was administered to all 2459 LHDs, the response rate was 61%. It is well known in the Profile study users’ community that the likelihood of nonparticipation is higher among LHDs with small population sizes. Given our findings that the food safety services are associated with population size, our results may have been affected by nonresponse bias, even after the use of statistical weights developed by NACCHO to account for the survey nonresponse. Secondly, the survey was self-reported, and the responses were not independently verified. Finally, the outcomes of interest representing food safety services were simply measured as dichotomous variables indicating whether each of those services was provided by LHDs or not. However, the variables did not measure the quality of the services or their scope comprising the details such as the number of people covered by the service, nature/type of those services, number of food establishments in the jurisdiction, and the extent of LHDs’ policy and advocacy involvement. Despite these limitations, the current study is useful in that it fills important gaps in the body of knowledge regarding the food safety activities carried out by LHDs, an area of research with existing evidence gaps. With a dearth of information on the impact of LHDs, this study sheds light on modifiable factors associated with the food safety, education, and policy engagement of LHDs that might assist with future planning and prioritization.

## 5. Conclusions

We live in an era where contemporary society comprises vulnerable groups such as the elderly, immunocompromised, and COVID-19-weakened long haulers, making food safety a critical but complex issue. Assessment and alignment of the public health workforce relative to public health service provision needs are critical, particularly for ensuring food safety and addressing poor health outcomes associated with foodborne illnesses. Our study highlights the essential role of nutritionists in food safety, showing that having at least one occupational nutritionist increased LHDs’ ability to engage in primary prevention activities, the inspection of food service establishments, and the provision of food safety education. Our study also suggests that LHDs, particularly those with local governance, may need capacity building and training and may benefit from resource-sharing related to food safety activities. Further, smaller LHDs may need capacity building, training, and provision of resources as a higher priority, given our findings that LHDs’ ability to provide food safety activities differed by their scope and scale, measured by the size of the population in their jurisdictions. Further, considering the looming threat of food-related bioterrorism, LHDs should be fully equipped and prepared to combat future food safety challenges since they are an integral part of our national food safety system. Any gains in Healthy People 2030 Safe Food Handling Objectives will depend on the optimal preparedness of LHDs to meet the existing and emerging food safety threats. Variations and gaps exist in LHDs’ engagement in food safety activities such as inspection of food processing and food service establishments, provision of food safety education, and primary prevention activities in nutrition. Factors associated with poor capacity for food service should guide intervention aimed at boosting LHDs’ food safety engagement because these activities are integral components of the Hazard Analysis and Critical Control Points (HACCP)—a systematic approach to the identification, evaluation, and control of food safety hazards. Resource availability may be the driving force for LHDs’ inclination to engage in food safety services and policy/advocacy activities. Efforts to address these gaps will benefit from LHDs’ collaboration with other community stakeholders that shape people’s equitable access to social determinants of health (SDoH) that positively impact food safety. LHDs’ superior policy engagement may require the adoption of the “Public Health Health 3.0” approach, wherein LHDs can serve as “chief health strategists” in influencing broader policies that shape food safety. In addition to capacity building, staff training, and budgetary provisions for food safety, LHDs that lack scope and scale may consider cross-jurisdictional resource sharing [40,41]. Food safety and nutrition play critical roles in the prevention and management of many chronic diseases; thus, training and budgetary priorities may be essential to assure LHDs will have a workforce specializing in food safety to deliver prevention activities targeted at foodborne risk reduction. A multidisciplinary approach that integrates food safety measures, basic nutrition education competencies in the public health studies curriculum, and in-service training must be made a top priority for LHD staff tasked with enhancing food safety and nutrition activities.

## Figures and Tables

**Table 1:** Descriptive Statistics for LHD characteristics. Profile of LHDs, 2019.

Variables	Frequency	% (Weighted)
**Population Categories**		
Less than 50,000	836	55.9
50,000–499,999	552	36.9
500,000+	108	7.2
Total	1496	100.0
**Governance Classification**		
State	315	21.1
Local	1043	69.7
Shared	138	9.2
Total	1496	100.0
**LHD has an Information System Specialist**		
No	1206	80.6
Yes	290	19.4
Total	1496	100.0
**LHD has registered, licensed practical or vocational nurse**		
No	91	6.1
Yes	1405	93.9
Total	1496	100.0
**Top certificate in Public Health**		
No	1118	79.1
Yes	295	20.9
Total	1413	100.0
**SP completed within 5 years**		
Yes, within 5 years	969	65.4
No, but plan to in the next year	144	9.7
No/Not within 5 years; no plan for next year	368	24.8
Total	1481	100
**LHD employs an occupational nutritionist**		
No	721	48.9
Yes	752	51.1
Total	1473	100.0
**LHD employs occupational environment health worker**		
No	359	24.4
Yes	1114	75.6
Total	1473	100.0
**CHIP completed within 5 years**		
Yes, within 5 years	1062	71.7
No, but plan to in the next year	124	8.4
No/Not within 5 years; no plan for next year	295	19.9
Total	1481	100.0
**CHA completed within 5 years**		
Yes, within 5 years	1176	79.4
No, but plan to in the next year	69	4.7
No/Not within 5 years; no plan for next year	237	16.0
Total	1482	100.0

Abbreviations: LHD—local health departments; CHA—community health assessment; CHIP—community health improvement plan; SP—strategic plan. NOTE: The table contains all independent variables considered for inclusion in the analysis.

**Table 2:** Logistic regression of food LHD activities for food safety inspections, education, primary prevention, and policy, 2019.

LHD Characteristics	Population-Based Primary Prevention Activity in Nutrition			Inspection of Food Processing Services			Inspection of Food Establishments			Food Safety Education			Policy and Advocacy Activities in Food Safety		
	AOR	95% CI		AOR	95% CI		AOR	95% CI		AOR	95% CI		AOR	95% CI	
		LL	UL		LL	UL		LL	UL		LL	UL		LL	UL
Size of LHD population (vs. Less than 50,000)															
50,000–499,999	1.2	0.9	1.8	1.2	0.9	1.5	2.6	1.9	3.6	1.7	1.3	2.3	1.7	1.4	2.2
500,000+	2.7	1.0	7.3	0.8	0.5	1.2	1.6	0.9	2.8	1.3	0.8	2.4	2.6	1.6	4.1
LHD governance (vs. state)															
Local	0.2	0.1	0.3	0.5	0.4	0.7	0.4	0.2	0.6	0.7	0.5	1.0	2.8	2.1	3.8
Shared	0.3	0.2	0.8	0.6	0.4	0.9	0.8	0.4	1.6	1.1	0.6	1.9	1.5	0.9	2.3
CHA completed within 5 years (vs. No/AND no plan in the next year)															
Yes, within 5 years	1.9	1.2	2.9	0.8	0.6	1.2	0.5	0.3	0.8	0.7	0.5	1.0	0.6	0.4	0.8
No but plan to in the next year	2.0	0.9	4.4	0.6	0.3	1.1	0.5	0.2	1.0	0.4	0.2	0.7	0.4	0.2	0.8
LHD employs a nutritionist (vs. No)															
Yes	20.0	12.4	32.2	1.1	0.9	1.4	1.4	1.0	1.8	1.4	1.0	1.9	0.8	0.7	1.1
A top executive has certification in Public Health (vs. No)															
Yes	0.6	0.4	0.9	0.9	0.7	1.2	1.2	0.8	1.7	1.2	0.8	1.6	0.9	0.7	1.1
LHD has a registered, licensed, or vocational nurse (vs. No)															
Yes	4.1	2.1	8.2	0.7	0.4	1.2	0.6	0.3	1.1	1.4	0.8	2.4	0.7	0.4	1.2

Abbreviations: LHD—local health departments; AOR—adjusted odds ratios; CI—confidence interval; LL—lower limit; UP—upper limit; CHA—community health assessment. Note: Table 2 presents the results of five different logistic regression models.

## Data Availability

Data used in this study are available at: https://www.naccho.org/resources/lhd-research/national-profile-of-local-health-departments#data-requests (accessed on 9 November 2021).
